# The Psychometric Properties of the Persian Version of the Contamination Cognition Scale (CCS)

**Published:** 2018-07

**Authors:** Zahra Zanjani, Hamid Yaghubi, Mohammadreza Shaeiri, Mohammad Gholami Fesharaki

**Affiliations:** 1 Department of Psychology, School of Medicine, Kashan University of Medicine Sciences, Kashan, Iran.; 2 Department of Psychology, School of Humanities, Shahed University, Tehran, Iran.; 3 Tarbiat Modares University, Tehran, Iran.

**Keywords:** *Contamination Cognition Scale (CCS)*, *Contamination*, *Obsessive-Compulsive Disorder*, *Psychometric Properties*, *Student*

## Abstract

**Objective:** In recent years, many researchers have been searching for effective cognitive factors in the development of obsessive-compulsive disorder (OCD). One of the scales designed to measure this characteristic is the contamination cognition scale (CCS) that evaluates 2 dimensions: overestimating the likelihood and severity of contamination. The aim of the present study was to evaluate the psychometric properties of the Persian version of CCS.

**Method** **:** The study population of this descriptive psychometric study included students of Shahed University. A total of 490 students were selected via cluster sampling and completed the CCS. CCS was translated and back- translated before given to the students. The Obsessive Beliefs Questionnaire (OBQ) and the Padua Inventory (PI) were used. To assess the evidence for the validity of the scale, the exploratory and confirmatory factor analyses were used. The gathered data were analyzed by SPSS-22 and Amos-22 software.

**Results: **The results of the confirmatory factor analysis (CFA) showed that one-factor model did not have adequate fitness (RMSEA>.05). Therefore, to explore the factors of this scale, exploratory factor analysis (EFA) was used, and it revealed 3 factors (public equipment, food, and restroom) for each of the dimensions (likelihood and severity). CFA by AMOS-22 confirmed the three-factor model (GFI, CFI, and NFI>.95; RMSEA<.05). Furthermore, the results supported criteria validity of CCS with the PI total score (0.56- 0.47, p<0.001) and PI-contamination subscale (0.71-0.75, p<0.001). Also, the correlation between CCS and responsibility/threat subscale of the OBQ was significant (0.47- 0.49, p<0.001) The Cronbach’s alpha for likelihood dimensions total was 0.93 and it was 0.94 for severity dimension total. The composite reliability was 0.95 for the likelihood dimension and 0.96 for severity dimension of CCS. Also, the test-retest reliability after a 4-week interval was confirmed (likelihood: r = 0.78; severity: r = 0.81, p<.001).

**Conclusion: **The results indicated that one-factor model of CCS did not have adequate fitness, but three-factor model was confirmed in both dimensions (likelihood and severity). According to the results of the present study, the reliability and validity of the Persian version of CCS were acceptable.

OCD is recognized as one of the most severe and chronic anxiety disorders (1). The most common type of OCD is contamination OCD (C-OCD), which is accompanied by washing behaviors or avoidance of the contaminated object. It has been reported that C-OCD is the most common OCD in Iran (2). Individuals with C-OCD who spend too much time for washing and cleaning are worried of becoming contaminated from dirt, germs, virus, or outside objects. These individuals always live with this fear that they are harming

themselves or others with these contaminations, or they cannot prevent the harm.

In response to these fears, they turn to excessive washing and bathing, or spend hours cleaning the house (3). 

It has been stated that the contamination/washing OCD has a stronger negative relationship with the quality of life compared to other types of OCD (4).

With respect to the etiology of this disorder, the Obsessive-Compulsive Cognitions Working Group (OCCWG) (5) Introduced 6 obsessive belief domains, based on which the cognitive models of OCD were constructed. These domains are as follow: 1) overestimation of threat: the tendency to overestimate the danger of situations, emotions, and mental events; 2) inflated responsibility: the belief that the person is responsible for preventing the harm; 3) perfectionism: the belief in doing everything perfectly; 4) intolerance of uncertainty: the belief that uncertainty is dangerous, and the person cannot tolerate it; 5) the importance of controlling one’s thoughts: the belief that thoughts can, and should be, controlled; 6) over-importance of thoughts: the belief in the importance of thoughts and in that thoughts can be harmful. 

Several studies have indicated that overestimation of threat rather than other beliefs had the stronger relationship with C-OCD (6-9). An experimental study about the perceived danger in a C-OCD group showed that the more the perceived danger, the more is the urge to clean (10).

One of the scales to measure the overestimation of threat is the responsibility/threat subscale of the Obsessive Beliefs Questionnaire (OBQ). Studies have shown that this subscale measures the threat overestimation in general and does not measure the threat overestimation about contamination, in particular, while C-OCD has cognitions about contamination and views the severity of the contaminant as rapidly growing (11). Researchers have presented some of the contamination-related cognitive factors in C-OCD, which can explain the compulsive behaviors and avoidance of the contaminated stimulants in C-OCD (12). Some scholars believe that these cognitions include the results of overestimation of the likelihood and severity of contamination. Individuals with C-OCD overestimate the likelihood (‘‘I will get sick if I don’t wash my hands’’) and severity (‘‘if I get sick, I will die’’) of contamination (7). The empirical evidence indicates that these cognitive factors are responsible for the onset of C-OCD (13).

Another scale that was developed to assess the overestimation of threat is CCS. Deacon and Olatunji (7) designed CCS to evaluate the tendency to overestimate the likelihood and severity of contamination. Unlike OBQ (the responsibility/ threat subscale) that measures the likelihood of threat and danger in general, CCS measures the overestimation of the likelihood and severity of threat of potential contaminated objects in particular. This scale evaluates 2 dimensions (severity and likelihood), each of which having 13 items. The total scores of CCS is the average of the 2 dimensions. Rating for the likelihood dimension ranges from 0 (not at all likely) to 100 (extremely likely) and for severity,

It ranges from 0 (not at all bad) to 100 (extremely bad). 

Deacon and Maack (14) have reported a good internal consistency (0.95- 0.99) and a test-retest reliability of 0.94 (p<.001) for CCS. Deacon and Olatunji (7) reported a correlation of 0.59 between this scale and the disgust scale (p<0.01). They reported good internal consistency for CCS (α =0.97). 

Eremsoy and İnözü (15) examined the psychometric properties of the Turkish version of the CCS and reported the Cronbach’s alpha of 0.89 and the test-retest reliability of 0.82 in a 4-week interval (p<0.001). Furthermore, the results indicated a good convergent validity for this scale with obsessive beliefs (r = 0.15- 0.36, p<.001) and trait anxiety (r = 0.15- 0.33, p<0.001). Also, CCS could discriminate people with low obsessive-compulsive symptoms from individuals with high obsessive-compulsive symptoms (14).

 CCS has been repeatedly used in studies on OCD and disgust (7, 15, and 16), and there are reports that the contamination cognitions are predictive of avoidance behaviors in individuals with fear of contamination (7). However, very few studies have been conducted on the psychometric properties of CCS. As a result, the aim of this study was to provide the Persian version of this scale and investigate its psychometric properties in Iranian population.

## Materials and Methods

***Participants and Study Design***

In this psychometric study, the study population was students of Shahed University. The sample was selected via cluster sampling. Considering that Comfrey and Lee (17) suggested a sample size of 300 individuals to study EFA and taking into account Myers et al.’s suggestion (18) of a sample size of 200 individuals for CFA, we selected a sample size of 500 university students. However, 490 individuals (156 male and 334 female students) fully completed the scales. Participants were randomly divided into two groups. CFA was conducted by a first half of the sample (n = 200), and EFA was performed on the second half of the sample (n= 190). 

***Measures***

1.  Contamination Cognition Scale (CCS)

This 26-item scale, which was designed by Deacon and Olatunji (7), included 13 items that OCD patients associated them with contamination (e.g., door handles, toilet seat). CCS assesses the overestimation of severity and likelihood of contamination. The total CCS items are 26, with each dimension (severity and likelihood) having 13 items. The participants are asked to imagine what would happen if they touched an object and were unable to wash their hands afterward. The participants must specify 2 ratings to each object: the likelihood that touching the object would cause contamination, and if contaminated, how bad would it be (7). The rating is based on a 0 to100 scale (zero = not at all likely, 50 = moderately likely, and 100 = extremely likely; or zero = not at all bad, 50 = moderately bad, 100 = extremely bad). Deacon and Olatunji (7) have reported good internal consistency for CCS (α =.97). They found a correlation of 0.59 between this scale and the disgust scale (p<.01). The psychometric properties of the Turkish version of the CCS were acceptable. The Cronbach’s Alpha was 0.89, and the retest reliability in a 4-week interval was good (r = 0.82, p<.001). This scale had the converge validity with OBQ-44 (r =0.15- 0.36, p<.001), and trait anxiety (r =0.15- 0.33, p<0.001) (15).

2.  Obsessive Beliefs Questionnaire (OBQ-44)

OCCWG developed the OBQ with 87 items, which was reduced to 44 items in later studies (12). This questionnaire was designed to examine 6 obsessive beliefs related to OCD. The OCCWG (19) reported a test-retest reliability of 0.95 for the total scale and 0.93 (p<.01) for the responsibility/threat subscales. 

The Persian version OBQ was examined by Shams et al. (20). Their results indicated that the Cronbach’s Alpha for the total scale and the responsibility/threat subscale was 0.92, and 0.85, respectively. The test-retest reliability after a 2-week interval was 0.82 (p<0.001) for the total scale and 0.78 (p<0.001) for the responsibility/threat subscale (18. In this research, factor analysis indicated 3 factors including, responsibility/threat (RT), perfectionism/certainty (PC), and importance/control of thoughts (IC). In this research, the RT subscale was used to study the convergent validity of the CCS (20).

3.  The Padua Inventory (PI)

This scale which consists of 60 items was developed by Sanavio (21). Each item is scored on a 5-point Likert from 0 (not at all) to 4 (very much). Sanavio has reported a 30-day interval test-retest reliability of 0.78 for men and 0.83 for women (19).

Goodarzi and Firoozabadi (22) confirmed the factor structure and reliability of the Persian version of the PI. 

*Adaption and Procedure*

To use CCS in Iran’s society, the following was done after the original copy of the scale was obtained:

1. The permission to use and translate the questionnaire was acquired from the authors of the scale (Dr. Deacon).
2. The scale was translated from English to Persian by a Ph.D. in clinical psychology, and then 3 psychology professors were consulted about the accuracy of the translation.
3. Five participants were invited to complete the questionnaire. These participants were then interviewed for suggestions to refine the readability, clarity, and comprehensibility of the instructions and items. They did not mention a problem with the instruction and clarity of items.
4. Then, 2 translators, who had not seen the original scale, translated it from Persian to English (back-translation).
5. The back- translated version was compared with the original version, and in case of any inconsistencies, the 2 translators were consulted to ensure the conceptual equivalence and the overall quality of the translation.
6. The back translated version was sent to the authors of the scale and was used after their approval.
7. The psychometric properties of the scale were studied. For this purpose, 500 individuals were selected among the University students by cluster sampling.
8. To examine the test-retest reliability, 47 of the participants were again tested after 4 weeks.
9. Then, the data were statistically analyzed.

***Statistical Analysis***

Data were analyzed by SPSS-22 and Amos-22 software. The EFA was conducted using the principal components analysis, and the oblimin rotation. The CFA was used to examine the model fit. To determine the reliability of CCS, Cronbach’s alpha and test-retest reliability (4-week interval) were used.

## Results

The participants consisted of 334 female (68.1%) and 156 male (31.8%) students aged 18 and 40 years (M = 21, SD = 4.01), and most of the participants were 18 and 21 years old (74.9%).

***Validity***

*Construct Validity*

To verify the construct validity of this scale, first, the proposed model of the creators of this scale was studied by CFA. Deacen and Olatunji (7) proposed 2 dimensions of severity and likelihood of contamination for this scale. They proposed one-factor model for each dimension. 

The results of the CFA of this model (Table 2) showed one-factor model for each dimension did not have adequate fitness (GFI, CFI, and NFI<.95; RMSEA>.05).

Therefore, to explore the factors of this scale, the EFA by principle component analysis was used. According to this analysis, the KMO coefficient was 0.93 and the X2 index for Bartlett’s test was 3724.09 (p<.001), indicating that the sample size and selected variables were adequate for factor analysis. The EFA by oblimin rotation revealed 3 factors for this scale. The correlation between these factors was above 0.30, indicating that the oblimin rotation was appropriate for factor analysis. 

According to the results of the EFA with oblimin rotation and factor loading of the items (Table 1), the first factor consisted of items 4, 5, 6, 7, and 8 in both dimensions of the scale. The second factor contained items 9, 10, 12, and 13, and the third factor included items 1, 2, and 3. The experts suggested that item 11 was removed because it was loaded on 2 factors in both dimensions and because of the uncertainty of this item for respondents. The final version of this scale, which measures 3 factors, contains 12 items in both likelihood and severity dimensions. Based on the content of the items, the factors were named “public equipment”, “food”, and “restroom”.

The correlation between the factors was above 0.3 (Table 2). As a result, employing the oblimin oblique rotation has been an appropriate method for analysis.

**Table1:** Results of the Exploratory Factor Analysis of CCS (n = 391)

**Dimensions**	**Estimation of the Likelihood of ** **Contamination**			**Estimation of the Severity ** **of Contamination**		
**Items**	**Factor ** **1**	**Factor 2**	**Factor 3**	**Factor 1**	**Factor ** **2**	**Factor 3**
1.Toilet handle in public restroom			0.69			0.76
2.Toilet seat in public restroom			0.89			0.86
3. Sink faucet in public restroom			0.58			0.68
4. Public door handles	0.78			0.74		
5. public workout equipment	0.78			0.83		
6. Public telephone receivers	0.91			0.83		
7. Stairway railings	0.90			0.83		
8. Elevator buttons	0.95			0.83		
9. Animals		0.60			0.63	
10. Raw meat		0.88			0.76	
11. Money	0.63	0.55		0.59	0.56	
12. Unwashed produce (eg, fruits, vegetables)		0.79			0.80	
13. Food that other people have touched		0.67			0.71	
Eigenvalue % of variance	8.02 34.50	1.11 23.48	1 20.05	7.6 34.17	1.16 21.07	1.07 20.51

Note. CCS: Contamination Cognition Scale

**Table2:** Correlation Coefficients between CCS Factors

**Dimensions**	**Factors**	**Factor 1**	**Factor 2**
Likelihood dimension	Factor 2	0.58	
	Factor 3	0.47	0.38
Severity dimension	Factor 2	0.55	
	Factor 3	0.45	0.38

Note. CCS: Contamination Cognition Scale

**Table3:** Results of the Confirmatory Factor Analysis of CCS Based on a Three-Factor Structure and One-Factor Structure

**Models**	**Goodness of fit indexes**	**X** ^2^ **/df**	**GFI**	**AGFI**	**CFI**	**NFI**	**RMSER**
	**Dimensions**						
Three-factor model	Likelihood dimension	1.88	0.96	0.93	0.99	0.98	0.04 (0.03- 0.06)
	Severity dimension	1.61	0.97	0.94	0.99	0.99	0.04 (0.02-0.05)
Single-factor model (proposed by Deacon and Olatunji)	Likelihood dimension	4.84	0.91	0.86	0.95	0.93	0.09 (0.08- 0.11)
	Severity dimension	5.01	0.89	0.83	0.94	0.93	0.10 (0.09- 0.11)

Note. CCS: Contamination Cognition Scale

**Table4:** Descriptive Indexes and Pearson’s Correlation between CCS, PI, the Contamination Subscale, and Responsibility/Threat Subscale of OBQ

**Dimensions**	**Factors**	**Mean ** **(SD)**	**PI Total**	**Fear of Contamination ** **Subscale**	**Responsibility/threat ** **Subscale**
Likelihood	Factor 1	52.79 (26.79)	0.50	0.48	0.44
	Factor 2	30.77 (24.39)	0.55	0.60	0.50
	Factor 3	49.53 (27.22)	0.48	0.65	0.36
	Total	44.37 (22.01)	0.56	0.75	0.47
Severity	Factor 1	59.49 (26.87)	0.53	0.65	0.45
	Factor 2	33.16 (26)	0.56	0.66	0.51
	Factor 3	48.68 (27.01)	0.53	0.64	0.41
	Total	47.11 (23.58)	0.47	0.71	0.49

Note. CCS: Contamination Cognition Scale; PI: Padua Inventory; OBQ: Obsessive Beliefs Questionnaire

**Table 5:** Results of the Test-Retest Reliability and the Cronbach’s Alpha for the CCS and Its Subscales

**Dimensions**	**Factors**	**Cronbach’s Alpha**	**Composite Reliability**	**Test-retest Reliability (n=47)**
Likelihood	Factor 1	0.95	0.96	0.84
	Factor 2	0.80	0.80	0.68
	Factor 3	0.85	0.84	0.71
	Total	0.93	0.95	0.81
Severity	Factor 1	0.80	0.83	0.79
	Factor 2	0.83	0.95	0.76
	Factor 3	0.88	0.86	0.69
	Total	0.94	0.96	0.78

Note. CCS: Contamination Cognition Scale

The findings of the CFA of the single-factor model showed that the X2/df index was >3, RMSEA was >.05, and the GFI, NFI, and CFI indexes were <.95, indicating the inadequacy of the model. However, all the goodness of fit indexes in the three-factor model were within the desired range. Consequently, confirmatory factor analysis supported the three-factor model of this scale (Table 3).

***Criteria and Convergent Validity***

To determine the criteria validity of the scale, its correlation with the PI and the PI contamination subscale and also the convergent validity with the OBQ (responsibility/threat subscale) were examined (Table 4). 

The results revealed that the correlation between the scale’s total score and PI was 0.56, it was 0.59 for the PI contamination subscale, and.51 for OBQ (responsibility/threat subscale) (P<.001).

***Reliability***

The test-retest reliability for the total scale was 0.80 after a 4-week interval (p<.001), and it was 0.81 and 0.78 for the likelihood and severity dimensions, respectively. The Cronbach’s alpha was 0.96 for the total scale, 0.93 for the likelihood dimension, and 0.94 for the severity dimension. Also, composite reliability was used to examine the reliability of the latent variable. Composite reliability is an ideal and alternative indicator for assessing the reliability of the variables in structural modeling, and it is more accurate than other methods of reliability evaluation, such as Cronbach’s alpha (23). Composite reliability was 0.95 for the likelihood dimension and 0.96 for severity dimension of CCS (Table 5). 

## Discussion

The aim of this study was to investigate the factor structure, convergent validity, and reliability of the Persian version of the CCS. To determine the factor structure of the CCS, first, one-factor model suggested by Deacon and Olatunji was examined by CFA (7). Concerning fit indexes of CFA, it has been stated that χ 2/df < 2 (24) and GFI, AGFI, TLI, and CFI >.9 is desirable. In the case of RMSEA, a value of less than 0.05 is considered as a good fit (25). Therefore, the CFA results showed that one-factor model did not fit well (X2/df >3, RMSEA>.05, GFI and CFI<.95). Then, EFA was used to extract the factors of CCS. The results of the EFA suggested 3 factors in both the likelihood and severity dimensions, which were named based on the contents of the items of each factor as “public equipment”, “food”, and “restroom”. Three factors of likelihood dimension explained 78.03% of the total variance and 3 factors of severity dimension explained 75.75% of the total variance. Item 11 (Money) was removed because it was loaded on 2 factors of “public equipment” and “food”. In addition, participants had difficulty answering it. The reason for this can be attributed to the cultural differences in understanding this item. It seems that the participants of the present study considered money as a material used by various individuals of the society; they also noted that the hands that are contaminated by this unclean money when touch any kind of food, can transfer the contamination to the individual. It is generally assumed that money can carry some microorganisms that cause food-borne disease and some researches have confirmed this belief (26). Hence, there is the possibility of cross- contamination between food and money, which can cause diseases. Therefore, it seems this belief has caused the item 11 (money) to be loaded in 2 factors. The results of the CFA indicated that the three-factor model, which consists of 12 items, had a better fit compared to the single-factor model. This finding was different from that of previous studies that suggested one-factor model for this scale (7, 14). Thus, we recommend 12-item version of CCS for the Iranian society that has 3 factors in each dimension.

 Regarding the criteria validity, it was found that the scale had a significant positive correlation with the total score of PI, as well as the PI contamination subscale. In addition, it was determined that the scale has a relationship with the responsibility/threat subscale. This finding was consistent with the opinion of Obsessive Compulsive Cognitions Working Group (5). As the literature refers to a relationship between the overestimation of threat and the OCD symptoms, this scale also had a relationship with the OBQ responsibility/threat subscale, which confirms the scale’s convergence. The finding of this study is consistent with previous studies (7, 14, and 15), indicating good validity for the scale. Furthermore, the results showed a good reliability of the scale based on Cronbach’s α (above 0.70) and test-retest reliability with 4-week interval. This result was consistent with previous research (14). Given the important role of overestimation of threat in the fear of contamination and obsessive-compulsive symptoms (5, 6, 9, and 13), this scale can help predict these symptoms. As there is not an adequate scale to assess threat overestimation of contamination in an Iranian population, this scale is recommended to be employed in research areas.

## Limitation

The limitations of this study were as follow: (1) The study sample was students, (2) the sample was non-clinical, therefore, restricting the generalization of results to other population, (3) this was a cross-sectional study and thus limited the inference of causal relationships, (4) limited questionnaires were used to assess the convergent validity of the scale, and thus it is recommended to use more questionnaires. Also, to generalize the results, it is recommended that next studies be conducted on clinical samples.

## Conclusion

To summarize, CCS has acceptable psychometric properties in Iran’s Persian speaking society, and it can be used with confidence to examine the overestimation of the contamination likelihood and severity of contamination. Given the relationship between contamination cognitions and obsessive-compulsive symptoms in previous studies and the present study, it seems that by assessing these cognitions we can identify individuals at risk of OCD.
